# Cost-utility analysis of bariatric surgery compared with conventional medical management in Germany: a decision analytic modeling

**DOI:** 10.1186/s12893-017-0284-0

**Published:** 2017-08-03

**Authors:** Oleg Borisenko, Oliver Mann, Anna Duprée

**Affiliations:** 1 Synergus AB, Danderyd, Sweden; 2 University Medical Center Hamburg, Hamburg, Germany

**Keywords:** Bariatric surgery, Cost-effectiveness, Cost-utility analysis, Germany

## Abstract

**Background:**

The objective was to evaluate cost-utility of bariatric surgery in Germany for a lifetime and 10-year horizon from a health care payer perspective.

**Methods:**

State-transition Markov model provided absolute and incremental clinical and monetary results. In the model, obese patients could undergo surgery, develop post-surgery complications, experience diabetes type II, cardiovascular diseases or die. German Quality Assurance in Bariatric Surgery Registry and literature sources provided data on clinical effectiveness and safety. The model considered three types of surgeries: gastric bypass, sleeve gastrectomy, and adjustable gastric banding. The model was extensively validated, and deterministic and probabilistic sensitivity analyses were performed to evaluate uncertainty. Cost data were obtained from German sources and presented in 2012 euros (€).

**Results:**

Over 10 years, bariatric surgery led to the incremental cost of €2909, generated additional 0.03 years of life and 1.2 quality-adjusted life years (QALYs). Bariatric surgery was cost-effective at 10 years with an incremental cost-effectiveness ratio of €2457 per QALY. Over a lifetime, surgery led to savings of €8522 and generated an increment of 0.7 years of life or 3.2 QALYs. The analysis also depicted an association between surgery and a reduction of obesity-related adverse events (diabetes, cardiovascular disorders). Delaying surgery for up to 3 years, resulted in a reduction of life years and QALYs gained, in addition to a moderate reduction in associated healthcare costs.

**Conclusions:**

Bariatric surgery is cost-effective at 10 years post-surgery and may result in a substantial reduction in the financial burden on the healthcare system over the lifetime of the treated individuals. It is also observed that delays in the provision of surgery may lead to a significant loss of clinical benefits.

## Background

Obesity is a serious disorder and is associated with an increased risk of developing diabetes [1, 2], cardiovascular disease [3–5], musculoskeletal diseases [6], gynecological problems [7, 8], and cancer [9]. Obesity is a rising epidemic in Germany, with 20% of the German population classed as clinically obese [10]. It is estimated that expenditure on obesity exceeds 17 billion Euros per year in Germany [10].

When conservative approaches to manage obesity fail, bariatric surgery represents the only effective method of weight reduction. The German society for general and visceral surgery (DGAV) guideline recommends bariatric surgery for patients with a body mass index (BMI) of >40 kg/m^2^ after failure of conservative management. However, in the presence of type 2 diabetes (T2D), the guideline recommends to lower the threshold to as low as BMI > 35 kg/m^2^ [11]. According to the Quality Assurance in Bariatric Surgery Registry, the number of procedures has increased from 1500 in 2006 to 5900 in 2011. The utilization of surgery in Germany is still relatively low compared with other European countries, with 72 bariatric surgeries per 1 million of the population in Germany, compared to 928 in Belgium, 571 in France, and 761 in Sweden [12].

Adoption of surgery is based on both the clinical and economic value for patients, the healthcare system, and society. To inform decision-makers about optimal criteria for bariatric surgery commissioning, it is important to study the cost-effectiveness of current surgical techniques used across the entire population of surgery candidates. This is also important for specific cohorts of patients with different severity levels of obesity, with and without pre-existing diabetes. Finally, this type of analysis can also be useful in evaluating the impact of a delay in the provision of surgery on health and economic outcomes from a public payer perspective. While the long-term clinical and economic consequences of bariatric surgery have been extensively studied in some European countries, the available studies for Germany include only one study published in 2006 [13]. This study aims to evaluate the cost-effectiveness of bariatric surgery in Germany from a statutory health insurance perspective over both mid-term (10 years) and lifetime horizons.

## Methods

**Fig. 1:**
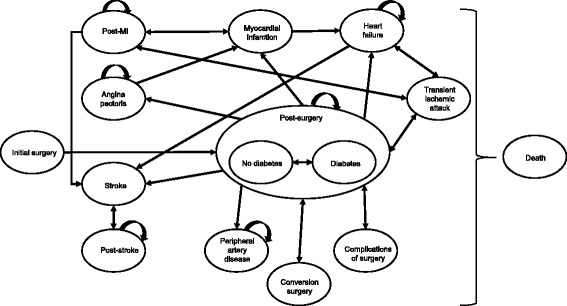
Structure of the model (reproduced from [15])

We used a state-transition decision analytic Markov model [14] to evaluate the cost-effectiveness of bariatric surgery compared with conventional medical treatment. Full details of the modeling approach, data inputs, and validation activities are reported elsewhere [15]. In brief, obese patients may be surgically operated on or continue with conventional medical management, experience post-surgery complications or have no complications, develop T2D or cardiovascular diseases (angina, myocardial infarction, stroke, heart failure and peripheral artery disease), recover from T2D or die (Fig. 1). In each model cycle, which is equal to 1 month, patients may either switch from one state to another or stay in the previous state. Cost-effectiveness was evaluated over 10-year and lifetime perspective.

## Input data

### Clinical effectiveness and safety data

The model predicts the risk of cardiovascular events, T2D, complications of surgery, and mortality in the population. The risk of cardiovascular events in the model is dependent on patient characteristics including age, gender, systolic blood pressure (SBP) measurements, BMI category, the presence of diabetes, and smoking status.

Data from previously published literature was used to estimate the risk of cardiovascular events [16, 17], diabetes incidence in relation to BMI [18, 19], remission of diabetes [20], the risk of short-term (30-day) mortality and severe adverse events [21–24], the risk of complications [24], and the rate of conversion surgery [25].

Age, gender, smoking status, BMI, SBP, and the presence of diabetes are risk factors for the development of obesity-related complications. Bariatric surgery leads to a reduction in the risk of obesity-related complications and mortality by reducing the risk of developing diabetes and decreasing BMI and SBP. Mortality data for the German population was acquired from gender-specific German life tables. Mortality due to ischemic heart disease (ICD-10 codes I20-I25) was subtracted from all-cause mortality to derive normal non-ischemic heart disease mortality. The presence of one of the cardiovascular events or diabetes leads to a higher risk of developing associated conditions (e.g. patients with heart failure are more likely to develop stroke) and mortality, which was informed by a number of epidemiological studies. Key clinical inputs are presented in Table 1.

**Table 1:** Clinical and cost inputs

Parameter	Value	Range	Distribution for probabilistic sensitivity analysis	Source
Patient baseline characteristic				
Age, years	40.4	25–65	Normal (SE = 4.04)	Stroh 2009 [23]
Gender, males (%)	26.3	-	Beta (α = 1315; λ = 3685)	Stroh 2013 [26]
Systolic blood pressure, mmHg	140.1	125–200	Gamma (α = 55.53; λ = 2.52)	Sjöström [20]
Body mass index, kg/m^2^	48.8	30–60	Normal (SE = 6.5)	Stroh 2013 [26]
Diabetes mellitus, (%)	20.6	-	Beta (α = 1030; λ = 3970)	Stroh 2013 [26]
Smoking, (%)	21.9	-	Beta (α = 1095; λ = 3905)	OECD factbook [52]
Cost inputs				
Cost of AGB	5621	4497–6745	-	G-DRG tariff K04B [40]
Cost of GBP and SG	8104	6483–9725	-	G-DRG tariff K04A [40]
Annual cost of T2D	3867	1934–7734	Gamma (α = 100; λ = 38.67)	Drabik 2012 [41]
Annual cost of acute stroke	4054	2027–8108	Gamma (α = 100; λ = 40.54)	Dodel 2004 (ratio of IS and HS is informed by Wolf 1992) [42] [43]
Annual cost of post-stroke 1 year	5493	2747–10,986	Gamma (α = 100; λ = 54.93)	Rossnagel 2005 [44]
Annual cost of post-stroke 2 year	6268	3134–12,536	Gamma (α = 100; λ = 62.68)	Kolominsky-Rabas 2006 [45]
Cost of transient ischemic attack	3327	2662–4159	Gamma (α = 100; λ = 33.27)	Dodel 2004 [42]
Cost of acute myocardial infarction	7644	3822–15,288	Gamma (α = 100; λ = 76.44)	Reinhold 2011 [46]
Annual cost of post-MI state	7056	3528–14,112	Gamma (α = 100; λ = 70.56)	Reinhold 2011 [46]
Annual cost of heart failure	5393	2697–10,786	Gamma (α = 0.34; λ = 15,982.32)	Peters-Klimm 2012 [47]
Annual cost of peripheral artery disease	2897	1449–5794	Gamma (α = 100; λ = 28.97)	Bruggenjurgen 2012 [48]
Annual cost of angina pectoris	3899	1950–7798	Gamma (α = 100; λ = 38.99)	Krankenhausdiagnosestatistik, 1997 [49] Statistisches Bundesamt Deutschland, 2005 [50]

*T2D* type 2 diabetes, *AGB* adjustable gastric banding, *GBP* gastric bypass, *MI* myocardial infarction, *CMM* conventional medical management, *SG* sleeve gastrectomy, *IS *ischemic stroke, *HS* hemorrhagic stroke, *SE* standard error

The model considered three of the most common surgical approaches: gastric bypass (GBP), sleeve gastrectomy (SG) and adjustable gastric banding (AGB). Distribution of different surgical methods for base-case analysis (GBP – 51%, SG – 17%, AGB – 33%) was obtained from the German Quality Assurance in Bariatric Surgery Registry [26].

The impact of different surgical methods on BMI was informed by the German Quality Assurance in Bariatric Surgery Registry in the base-case analysis [26]. Beyond the latest observation (1 year), impact on BMI was extrapolated using BMI change data from the SOS (Swedish Obesity Subjects) study [27]. After 15 years, BMI level was assumed permanent until death. The effect on BMI in the conventional medical management arm was assumed to be equal to the rate of BMI reduction in the control arm of the SOS study [27]. The change in SBP was informed by the SOS study for non-diabetic [20] and a study for diabetic [28] patients.

A number of individual studies [25, 29–37] informed the change in BMI for analysis in different individual cohorts of patients. Where standard deviation was not reported, it was obtained from Nguyen et al. [25].

Health-related quality of life was related to BMI level and the presence of diabetes [38]. The disutility of cardiovascular disease was also considered [39].

### Resource utilization and cost data

Evaluation of resource utilization data with cost, as well as the number of surgical procedures and prevalence of surgical methods, was performed using German sources. Only direct medical costs were included in the analysis.

The costs of end-stage organ damage health states were also obtained from the German G-DRG tariffs available [40] and German literature [41–50].

It was considered that pre-operatively a patient would have two consultations with the surgeon, one visit to a dietician and a psychologist. After surgery, two visits to a surgeon and nurse were considered during the first month, followed by one visit to the surgeon during the first year. From the second year, only one annual consultation by a dietician was considered. In the medical management arm, one annual general practitioner (GP) and dietician visits were assumed.

Cost data are presented from 2012 in Euros (Table 1). Inflation adjustment was performed using the German consumer price index [51].

### Cohort description

The model took into consideration two types of patient cohorts. First, multiple cohorts with characteristics informed by average values and distributions extracted from German studies [23, 26], SOS study [20], and OECD data about the proportion of the population who smoke [52] (Table 1). Second, the cost-effectiveness of bariatric surgery was estimated in 16 cohorts of 41-years old non-smoking males and females with severe (start BMI – 33 kg/m^2^), moderate (start BMI – 37 kg/m^2^), morbid (start BMI – 42 kg/m^2^), and super obesity (start BMI – 52 kg/m^2^), with and without the presence of T2D.

### Analysis

The incremental cost-effectiveness ratio (ICER) was calculated by comparing the difference in average total costs with the difference in average quality-adjusted life-years (QALY) among the study cohorts. All costs and outcomes beyond the first year were discounted 3.0% annually, according to the German recommendations [53].

Apart from the standard analysis of cost-effectiveness between two technologies, we performed analysis of the impact on the delayed provision of surgery vs. immediate provision on clinical and economic outcomes in a cohort of non-diabetic patients. Patients were initially present in the conventional medical management arm and were modeled to move to the surgical arm after 1, 2 and 3 years. Results were compared with the analysis in which patients receive surgery immediately.

The model was constructed using Microsoft Excel 2013 (Microsoft Corp., Redmond, Washington, USA).

### Sensitivity analysis

The sensitivity of the model was assessed by determining how the output changed when input parameters were altered. This was performed using a one-way sensitivity analysis over the lifetime horizon. Variables were adjusted one at a time, within a predetermined range, while the remaining parameters were unaltered. The analysis was performed for a single cohort of 40.4 years old males with a BMI of 48.8 kg/m^2^, with non-smoking and diabetes-free status. Specific conditions were applied to the binary input parameters (gender, smoking and diabetes status). For the “gender” parameter, “male gender” was considered as maximum input (value of 1), “female gender” as minimum input (value of 0). For diabetes and smoking, their presence was considered as maximum input (value of 1), their absence as minimum input (value of 0).

To examine the simultaneous uncertainty around all parameters in the cost-effectiveness analysis, multivariate probabilistic sensitivity analysis (PSA) was applied with 5000 iterations for each estimation. Key input parameters in the deterministic analysis that were assumed to be random variables were baseline patient characteristics, costs, utility decrements, probabilities and relative risks.

When cost estimates were available only as single-point estimates, they were assumed to follow a γ distribution. Reimbursement tariffs were not tested in PSA. A β distribution was assigned to the probabilities, utility decrements and a log-normal distribution to the relative risks. A normal distribution was assigned to the patient age and BMI, while SBP was assumed to follow a γ distribution. Results from the PSA are presented through cost-effectiveness acceptability plane, which graphically assesses the boundaries of the incremental costs and clinical gains.

## Results

### Model validation

The model was extensively validated both internally, to assess the technical performance of the model, and externally, for comparison of the outcomes with results from the ASCOT-BPLA [54], AHEAD [55] and ACCORD [56] studies, and the Scandinavian Obesity Surgery Registry [57] validation, for which results are reported elsewhere [15]. External validation showed that the model predicts the majority of clinical events (cardiovascular mortality, stroke, health failure, angina, peripheral artery disease, diabetes incidence, and remission) with a high degree of precision, although there was a tendency to overestimate all-cause mortality and combined (fatal and non-fatal) myocardial infarction.

### Base-case results in multiple cohorts extrapolated from German quality Assurance in Bariatric Surgery Registry

In the base-case analysis at 10 years, bariatric surgery was associated with higher costs of €2909, an additional 0.03 life years, and 1.2 QALYs, which resulted in an incremental cost-effectiveness ratio of €2457 when compared to conventional medical management. In the base-case analysis over the lifetime of the patient cohort, bariatric surgery led to cost savings of €8522 and generated an additional 0.7 life years and 3.2 QALYs (Table 2). Surgery is a dominating alternative to conservative management, as it is both more effective and less expensive.

**Table 2:** Results of cost-effectiveness analysis

	Cost, €	∆ cost	LYG	∆ LYG	QALY	∆ QALY	ICER, €/QALY
10 years							
CMM arm	11,501	2909	8.6	0.03	3.3	1.2	2457
Surgical arm	14,410		8.6		4.5		
Lifetime							
CMM arm	49,107	−8522	20.8	0.7	7.5	3.2	Dominates
Surgical arm	40,585		21.5		10.7		

*ICER* incremental cost-effectiveness ratio, *LYG* life years gained, *CMM* conventional medical management, *QALY* quality-adjusted life years

In the simulation, surgery led to a substantial reduction in lifetime risk of obesity-related adverse events (Table 3): from an 11% reduction in the risk of transient ischemic attack to a 29% decrease in the incidence of T2D. It was observed that surgery had the potential to reduce the risk of obesity-related adverse events significantly, for both 10 years and lifetime horizons (Table 3).

**Table 3:** Absolute and relative risks of obesity-related adverse events in the model

	Angina	MI total non-fatal	MI fatal	Stroke total non-fatal	Stroke fatal	TIA	HF	PAD	T2D
10 years									
Absolute risk in surgical arm	0.02	0.04	0.0006	0.02	0.0040	0.003	0.03	0.02	0.12
Absolute risk in CMM arm	0.03	0.06	0.0010	0.03	0.0056	0.005	0.03	0.03	0.23
Relative risk	0.72	0.72	0.64	0.73	0.72	0.73	0.73	0.73	0.53
Lifetime									
Absolute risk in surgical arm	0.12	0.25	0.02	0.19	0.03	0.02	0.17	0.10	0.33
Absolute risk in CMM arm	0.13	0.29	0.03	0.23	0.04	0.02	0.19	0.12	0.47
Relative risk	0.87	0.85	0.77	0.84	0.84	0.89	0.86	0.89	0.71

*HF* heart failure, *MI* myocardial infarction, *PAD* peripheral artery disease, *TIA* transient ischemic attack, *CMM* conventional medical management, *T2D* type 2 diabetes

### Results in specific cohorts of patients

When the modeling approach was applied in individual cohorts, bariatric surgery was cost saving over a 10 years horizon in all eight diabetic cohorts considered (moderately, severely, morbidly and super obese males and females). In non-diabetic cohorts, surgery was cost-effective in all cohorts, moderately obese males (ICER €9835/QALY) and females (ICER €10,436/QALY), severely obese males (ICER €8019/QALY) and females (ICER €8451/QALY), morbidly obese males (ICER €4048/QALY) and females (ICER €4320/QALY), and super obese males (ICER €1880/QALY) and females (ICER €2016/QALY). The cost-effectiveness of surgery was driven by a higher baseline BMI level of the cohort.

Over a lifetime horizon, bariatric surgery was also cost-saving in all eight diabetic cohorts considered. In non-diabetic cohorts, surgery was cost saving in all cohorts, except moderately obese males (ICER €1171/QALY) and females (ICER €523/QALY), and severely obese males (ICER €377/QALY). Surgery remained very cost-effective and was well below the assumed ‘willingness-to-pay’ threshold of €35,000/QALY.

### Impact of waiting lists on clinical and economic benefits of bariatric surgery

**Fig. 2:**
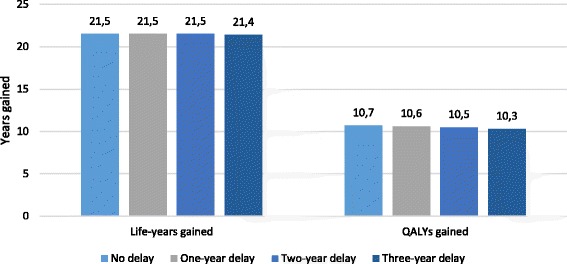
Life years and QALYs gained with performing surgery immediately and with a delay

It was also demonstrated that a delay of surgery for up to 3 years led to a significant loss of clinical benefit. The 3-years delay was associated with differences of 0.1 life years gained and 0.4 QALYs compared to immediate operation (Fig. 2). The cost for provision of surgery decreased with the delay of surgery provision, compared with the cost of immediate surgery provision. The cost of the surgery over the lifetime horizon accounted for €40,585 with the immediate operation, €40,152 with a 1-year, €39,514 with 2-year, and €39,841 with a 3-year delay.

### Deterministic sensitivity analysis

Baseline results for deterministic sensitivity and scenario analyses include cost-saving effect over a lifetime, with a cost impact of € 2494, gain of 0.9 life years, and 2.9 QALYs.

**Fig. 3:**
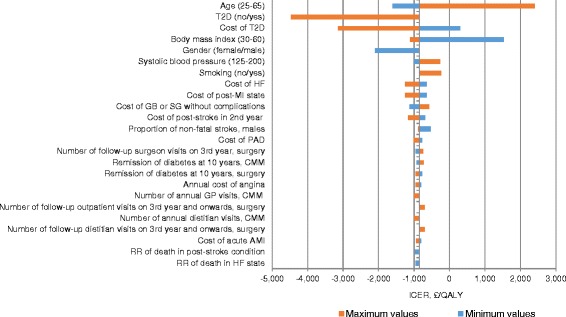
Tornado diagram. The figure shows one-way sensitivity analysis at lifetime horizon. The figure presents maximum and minimum values effect in ICER €/QALY. Parameters that affect results for more than €100 were included into the graph

Base-case scenario for deterministic one-way sensitivity analysis showed that three parameters could affect the cost-saving effect of surgery (surgery becomes cost-effective): start age (towards increase of age), cost of treatment of T2D (towards lower costs of treatment), and BMI (towards decrease of start BMI) (Fig. 3). Change of cost variables by 50% from the mean did not influence cost saving effect of surgery except for the cost of type II diabetes.

In general, extensive sensitivity and scenario analyses showed that the uncertainty around model inputs and structure does not affect the main results significantly.

### Probabilistic sensitivity analysis

**Fig. 4:**
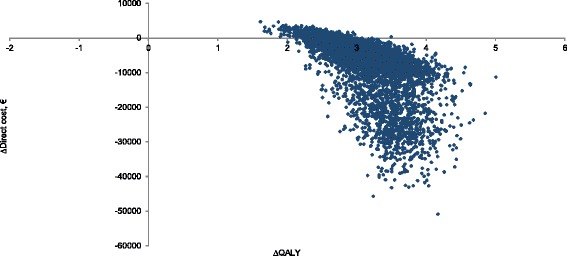
Cost-effectiveness acceptability plane. The figure shows the distribution of 5000 Monte Carlo simulations at a lifetime horizon. In the figure two populations are presented, which differ by the presence of type 2 diabetes at the start of the model (diabetic patients have a higher level of cost-saving)

The probabilistic sensitivity analysis with 5000 Monte Carlo simulations over lifetime horizon demonstrated that bariatric surgery produces clinical benefits (additional QALYs) in all patients, and has a cost-saving effect in 92.3% of cases, while in the remaining 7.7% it is cost-effective (Fig. 4).

## Discussion

The results of our analysis, driven by a mix of currently used surgical techniques, imply that bariatric surgery is cost-effective over the period of 10 years. It has a cost-saving effect on the health care system over a patient’s lifetime, in a German setting, and is associated with substantial clinical benefits.

As the types of surgery differ in the efficacy and consequent short and long-term costs, the resulting incremental costs, clinical gains, and ICER are dependent on the current treatment mix. Thus, GBP might be associated with the most beneficial economic outcomes. However, this was not specifically studied in the current analysis.

When the demonstrated lifetime clinical and economic benefits are applied to the cohort of German patients, who received surgery in 2011 (*n* = 5613), this results in savings of about €47.8 M and generates an additional 3929 person-years or 17,962 QALYs. The cost-saving effect was also demonstrated over a lifetime for patients with pre-existing T2D and patients with morbid and super obesity irrespective of the presence of diabetes.

To our knowledge, this is one of the first health economic evaluations of bariatric surgery in Germany. Another identified publication showed results of a decision analytic model used to perform cost-effectiveness and budget impact analyses of GBP and AGB over conventional treatments in three European countries [13]. The results of our analysis are in agreement with the results of the study [13], which has shown that surgery has a cost-saving effect and increases QALY. Our analysis demonstrated that operative obesity treatment was cost-saving in Germany and lead to an additional 1.34 QALY with GBP and 1.03 QALY with AGB over the base case time horizon of 5 years. The study has several assumptions that differ from our analysis and make a further direct comparison of the results difficult, including evaluation of either GBP or AGB one at a time, short time-horizon, and a specific patient population (BMI > 35 kg/m^2^ and diabetes type 2). Our analysis incorporated three of the most common bariatric surgical techniques adopted a short-term (10 years) and lifetime horizon and was focused on two types of cohort including those with patient characteristics taken from German studies.

Our study attempted to quantify the potential impact of extensive waiting lists on cost and clinical outcomes of bariatric surgery. It was shown that a 3-year delay in surgery provision might slightly reduce the total cost of treatment. At the same time, a delay in surgery may lead to loss of clinical benefits up to 0.1 life-years and 0.4 QALYs over a lifetime. While this indicates the importance of a reduction in waiting times, it is not a well-studied area [58–60]. The results presented here highlight the necessity to combat waiting lists and to remove unnecessary barriers before surgery for patients.

Despite wide acknowledgment of bariatric surgery’s short and medium-term effectiveness and cost benefits, operative treatment in Germany is underutilized. Total per capita spending on bariatric surgery is at least 7 times lower than in some other European countries such as Belgium, Sweden, and France [12]. One potential reason for this is that reimbursement of these procedures in the German healthcare system is complicated by a restrictive regulation, performed by health insurance companies. Operative obesity treatment can be accepted only after individual medical expertise and is not always approved [61]. Referral pathways should be enhanced, and clearly defined conservative treatment criteria should be established before a wider adoption of bariatric surgery in Germany.

The study has some limitations, which were discussed in detail elsewhere [15]. In brief, the analysis did not account for all potential obesity-related complications and potentially underestimated cost benefits from the surgery. The model used here did not distinguish outcomes of the surgery on different populations of diabetic patients, which could affect the overall clinical effectiveness of the therapy. The data on the management of post-surgical patients or surgical candidates who do not undergo surgery was driven by assumptions based on clinical knowledge.

Generally, a model-based approach is a simplification of reality. Validation of our model revealed that it overestimates all-cause mortality and myocardial infarction, however, estimates from validation studies were within a credible interval of model estimates. In addition, the impact of surgery on cardiovascular events might be overestimated taking into account change of medical practice over the last two decades (e.g. the introduction and widespread use of statins).

## Conclusions

Bariatric surgery is cost-effective at 10 years and may lead to significant cost-savings to health care system over the lifetime. Delay in the provision of surgery may result in significant losses of clinical benefits.

## Abbreviations

BMI: Body mass index
DGAV: Deutschen Gesellschaft für Allgemein- und Viszeralchirurgie (The German Society for general and visceral surgery
GB: Gastric banding
GBP: Gastric bypass
ICD: International classification of diseases
ICER: Incremental cost-effectiveness ratio
OECD: The Organization for Economic Co-operation and Development
QALY: Quality adjusted life-years
SBP: Systolic blood pressure
SG: Sleeve gastrectomy
T2D: Type II diabetes mellitus
